# Engineering transparency: a conversation with Guosong Hong

**DOI:** 10.1117/1.BIOS.3.2.020501

**Published:** 2026-06-26

**Authors:** Darren Roblyer, Travis Sawyer

**Affiliations:** a Boston University, College of Engineering, Boston, Massachusetts, United States; b University of Arizona, Wyant College of Optical Sciences, Tucson, Arizona, United States

## Abstract

BIOS Editor-in-Chief Darren Roblyer and Associate Editor Travis Sawyer interviewed Guosong Hong about his path in biophotonics.

**Figure:**
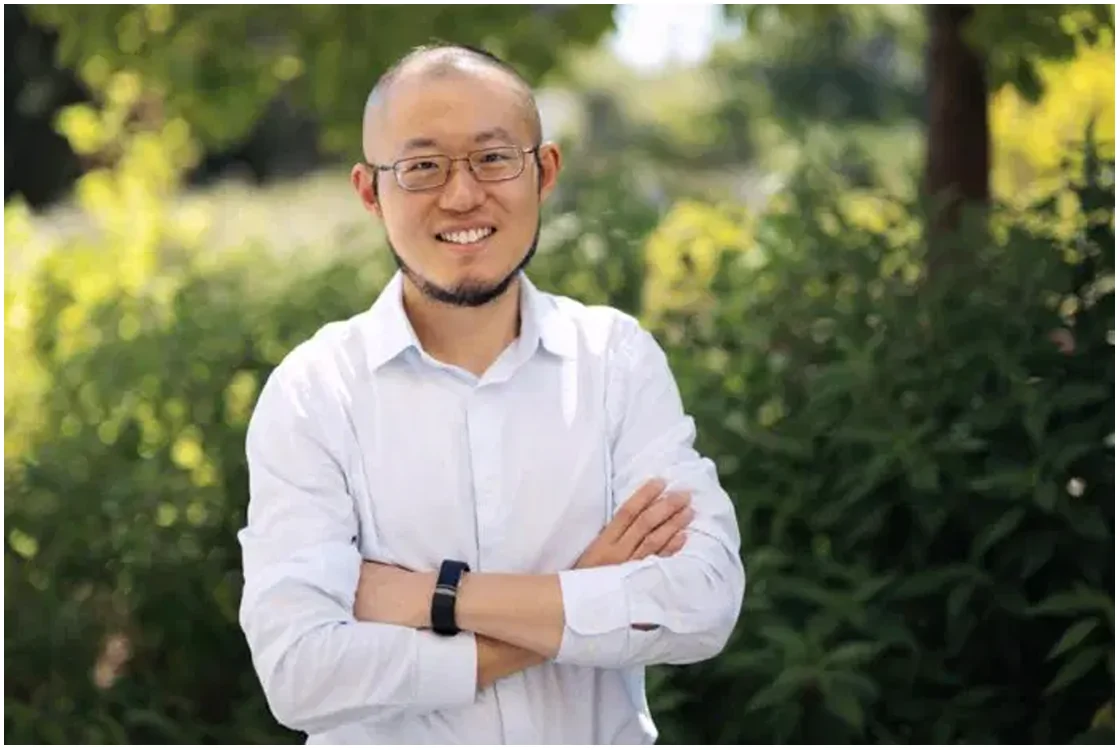
*Guosong Hong, Assistant Professor of Materials Science and Engineering at Stanford University, and inaugural recipient of* Biophotonics Discovery’s *Impact of the Year Award. Listen to our interview at* *Biophotonics Discovery: The Podcast*.

Guosong Hong, Assistant Professor of Materials Science and Engineering at Stanford University, is the inaugural recipient of *Biophotonics Discovery*’s Impact of the Year Award, which recognizes recent contributions that have made a meaningful impact on the field. Hong was honored by the journal community for his pioneering work in optical clearing—demonstrating that biological tissues in live animals can be made optically transparent. First presented at SPIE Photonics West 2025 and later published in *Science*, the work has already been reproduced in more than 20 independent studies.

Hong’s path into biophotonics began during his PhD at Stanford, where he focused on *in vivo* fluorescence imaging in the second infrared window, a wavelength range where light scattering is reduced and imaging depth improves. With a background in chemistry, he developed biocompatible carbon nanotubes and quantum dots that emit in this range, enabling noninvasive imaging of brain vasculature and blood flow in mice. After a postdoctoral fellowship at Harvard developing neural recording tools, Hong returned to optics when launching his own lab at Stanford.

A key insight behind the optical clearing work emerged during the COVID-19 pandemic, when Hong’s group revisited fundamental texts on light scattering. By reexamining how absorption and scattering are linked through refractive index, the team realized that tuning absorption could be used to engineer scattering itself. This led to a general strategy: introducing strongly absorbing molecules into tissue to reduce scattering and increase transparency.

The implications extend broadly across biology and medicine. Tissue opacity limits almost all optical techniques, and reducing scattering enables deeper, clearer imaging without changing imaging hardware. Importantly, the effect is not limited to a single compound. A range of absorbing molecules can serve as clearing agents, including FDA-approved dyes such as fluorescein and indocyanine green.

One of the major challenges, Hong noted, was identifying molecules with the right spectral properties. Many dyes absorb too broadly, negating the desired effect. Candidates must absorb strongly at a target wavelength while dropping off quickly beyond it. Tartrazine, fluorescein, and indocyanine green meet these criteria and emerged as effective options through database screening and experimental validation.

Looking ahead, Hong and collaborators are working to translate the approach to human applications, including less invasive cancer diagnosis through skin imaging and restoring transparency in corneal and lens tissues. Beyond optical clearing, Hong’s lab is also developing mechanoluminescent nanoparticles that convert focused ultrasound into light inside the body. Injected into the bloodstream, the particles can be activated noninvasively to emit light at specific locations, enabling applications such as optogenetics and gene editing without implanted optical fibers.

Asked what advice he would give to young researchers, Hong emphasized the value of foundational knowledge. “Go to the old books,” he said. “Many deep ideas are already there, waiting to be rediscovered in the right context.”

## Supplemental Information

The full interview with Guosong Hong is available in podcast form at *Biophotonics Discovery: The Podcast*, available wherever you get your podcasts.

